# Elevated temperature exacerbates red sore disease caused by *Vibrio vulnificus* in American eels (*Anguilla rostrata*)

**DOI:** 10.3389/fmicb.2026.1911288

**Published:** 2026-10-05

**Authors:** Amanpreet K. Kohli, Hannah N. Brown, Catharine R. Cipriano, David T. Gauthier, Wolfgang K. Vogelbein, Andrew R. Wargo

**Affiliations:** 1 Virginia Institute of Marine Science, William & Mary, Gloucester Point, VA, United States; 2 North Carolina Sea Grant, North Carolina State University, Raleigh, NC, United States; 3 Old Dominion University, Biological Sciences, Norfolk, VA, United States

**Keywords:** *Anguilla rostrata*, inflammation, Koch’s postulates, red sore disease (RSD), stress, temperature, *Vibrio vulnificus*

## Abstract

The marine pathogenic bacterium *Vibrio vulnificus* is linked to epizootics of a dermal ulcerative disease, causing high mortality in European and Japanese eels. American eels (*Anguilla rostrata*) experience a similar condition, commonly referred to as red sore disease (RSD). Field studies indicate that *V. vulnificus* infections highly correlate with RSD in American eels, but causation remains un-established. In this study, clinically healthy American eels were intraperitoneally injected with *V. vulnificus* and then held for 12 days at 18, 25, or 32°C. Presence of characteristic clinical signs, mortality, and consistent bacterial re-isolation fulfilled Koch’s postulates for *V. vulnificus* as an etiological agent of RSD in American eels. Gross clinical signs included hyperemia, hemorrhage, epidermal erosion, and shallow to deep ulceration with sub-dermal tissue necrosis. Histopathology indicated widespread colonization by Gram negative bacteria; partial to complete loss of epidermis and dermis (including scales); as well as edema, hemorrhage, myonecrosis, and extensive leukocytic infiltration (inflammation) of affected tissues. Eels injected with *V. vulnificus* had 92 times higher odds of death than controls. Elevated temperature was an additional risk factor with the odds of *V. vulnificus*-injected eels dying being 13 times greater at 32°C compared to 18°C. This directly related to the increased growth of *V. vulnificus* at higher temperatures observed *in vitro*. These findings identify temperature as an important driver for RSD risk in wild American eel populations and a mitigation focus for eel aquaculture in the Chesapeake Bay region of the United States.

## 1 Introduction

An emerging infectious disease, commonly referred to as red sore disease (RSD), has recently been reported from the American eel (*Anguilla rostrata*) aquaculture industry in Virginia, United States (Kohli et al., 2025). Eel potters in the Chesapeake Bay region are experiencing increased prevalence of RSD in their temporary holding facilities and incur high mortalities attributed to this condition. Eel potters have also seen RSD in wild eels (pers. comm. with Chesapeake Star Seafood and 2 Barrels Baits), with similar clinical signs observed in other wild finfish populations of the Southeastern United States (Reed and Francis-Floyd, 1993; Ksepka and Bullard, 2021). Dermal RSD pathology in American eels, as observed by Kohli et al. (2025), includes hyperemia, widespread hemorrhage, loss of scales, erosion of epidermis, and superficial to deep necrotic skin lesions that expose underlying muscles or bones. Noga and Berkhoff (1990) reported a similar ulcerative skin disease causing morbidity in wild and cultured populations of American eels in North Carolina, United States. Studies from the 1980s in North American eel aquaculture indicated *Aeromonas hydrophila* and *Aeromonas salmonicida* as the etiological agents of RSD (Hayasaka and Sullivan, 1981; Inman and Bland, 1981; Davis and Hayasaka, 1983; Noga and Berkhoff, 1990).

Recently, we found RSD in aquaculture to be associated with *Vibrio* spp. in addition to aeromonads, with *Vibrio vulnificus* indicated as being dominant in severe and systemic infections (Kohli et al., 2025). These findings are consistent with disease reports from eel aquaculture in Europe and Japan, where a clinically similar disease was determined to be caused by *Vibrio* spp. (primarily *V. vulnificus*) infection in *Anguilla anguilla* and *Anguilla japonica* (Muroga et al., 1976; Dalsgaard et al., 1999; Fouz et al., 2006; Elgendy et al., 2016). Despite strong clinical and epidemiological associations, the causal link between *V. vulnificus* and RSD for the recent American eel disease outbreak in the Chesapeake Bay of North America, has not been directly established through controlled laboratory experiments.

*Vibrio* spp. are opportunistic pathogens and RSD due to bacterial infections in finfish is commonly associated with poor water quality (Noga, 2010). Under stressful conditions, localized skin infections by bacteria such as *Vibrio* spp. can turn systemic, causing necrosis of internal blood filtering organs and driving acute mortality (Noga, 2010). Temperature, in particular, has been found to be associated with increased eel mortality due to *Vibrio* spp. infections (Amaro et al., 1995). Further, we previously observed that in American eel aquaculture operations, the odds of detecting *Vibrio* spp. increased with temperature such that it became the dominant bacterial pathogen above 25°C (Kohli et al., 2025). Taken together, these findings suggest that RSD in American eels is driven by an interaction between temperature and a *Vibrio* spp. infection. Specifically, increasing temperature may not only enhance replication and prevalence of *Vibrio* spp. in the host and the environment (Marco-Noales et al., 1999; Randa et al., 2004; Hernández-Cabanyero and Amaro, 2020), but may also stress eels (Barila and Stauffer, 1980; Tzeng et al., 1998; Jessop, 2010), thereby exacerbating disease pathology.

We conducted *in vivo* laboratory exposures, using American eels (*Anguilla rostrata*) as experimental animals, to: (1) confirm *Vibrio vulnificus* as a causative agent of RSD by applying Koch’s postulates (Koch, 1884) and (2) assess the role of temperature on clinical severity of RSD. Briefly, healthy eels were inoculated with a *V. vulnificus* isolate collected from an eel exhibiting RSD in the field. The inoculated eels were then held under different temperatures to monitor disease progression, with infection confirmed via pathogen re-isolation (Grimes, 2006), sequencing, and histology (Noga, 2010). *In vitro* pathogen growth was also measured across temperatures to gain insights into how disease severity was influenced by the direct effects of temperature on eels relative to pathogen replication.

## 2 Materials and methods

### 2.1 Bacterial isolate

A *Vibrio vulnificus* isolate (herein labeled AK-2020-508B) was recovered from an eel harvested by a commercial eel potter from the York River, Virginia in 2020. The fish showed mild clinical signs of RSD and harbored a systemic infection (i.e., liver and spleen). The bacterium was originally aseptically collected from the spleen of the animal, following methods outlined in Kohli et al. (2025), and isolated by culturing at 27°C for 48 h on a tryptic soy agar (TSA) plate. A single opaque colony was isolated from the culture plate and then re-inoculated into a test tube with 10 mL of Tryptic Soy Broth (TSB) and allowed to grow for 6–8 h at 27°C in an orbital incubator at 120 rpm. A 500 μL volume of culture was diluted with glycerol at a final concentration of 25% (vol/vol) and then stored at −80 °C for future analysis.

### 2.2 Molecular identification of bacteria

The isolate AK-2020-508B was revived from the −80 °C glycerol stock on a (TSA) plate at 32°C with a 24–48 h incubation period. An isolated colony was used to further incubate 10 mL of TSB in a test tube for 6 h. Bacterial DNA was extracted from 2 mL of this culture by using a DNeasy^®^ Blood and Tissue Kit (Qiagen), following the manufacturer’s directions. Briefly, the culture was centrifuged, and the pellet resuspend in PBS buffer, followed by lysis with proteinase K. Through another centrifugation, the lysate was bound to the column membrane, and contaminants and enzyme inhibitors were washed away with buffers AW1 and AW2 containing ethanol. Purified DNA was eluted in 200 μL water with two subsequent centrifugation steps each containing 100 μL of elute.

Extracted DNA was prepared for Illumina sequencing with a KAPA Hyperplus kit (Roche Diagnostics, Indianapolis, IN) and KAPA Unique Dual Index adapters. The library was sequenced using an Illumina MiSeq with the nano v2 2 × 250 sequencing kit (Illumina, San Diego, CA) to an approximate mean depth of 67×. Reads were reference-assembled to *Vibrio vulnificus* YJ016 (Genbank #s NC_005139, NC_005140, NC_005128) using the reference assembly tool in Geneious v. 2019.1.3 (Kearse et al., 2012).

### 2.3 *In vitro* growth kinetics of *V. vulnificus*

An *in vitro* study was carried out to explore the growth kinetics of *V. vulnificus* isolate AK-2020-508B as a function of temperature and salinity. The isolate was revived from the −80 °C glycerol stock on a (TSA) plate at 32°C with a 24–48 h incubation period. Single colonies were collected and then suspended in 50 mL of TSB in 125 mL flasks (one colony per flask) and incubated in orbital incubators at 120 rpm. The liquid cultures were grown at three different salinities (0, 10, and 20 ppt made using Instant Ocean Sea Salt^®^) and three different temperatures (20, 27, and 35°C) in a fully factorial design, to give 9 total culture flasks. Optical density (OD_600_) of each culture was measured every hour for 7 h, as a proxy for bacterial growth in the liquid culture (Ram et al., 2019; Beal et al., 2020). TSB with no bacterial inoculation was used as a blank control. To confirm the relationship between OD_600_ and colony forming units (CFU), a 100 μL culture of AK-2020-508B grown at 35°C and 10 ppt salinity was also sampled at each time point, serially diluted (10^–1^ to 10^–8^), and grown on TSA plates at 35°C for 24 h (Supplementary Figure 1). Two additional isolates of *V. vulnificus* (AK-2020-515C, and AK-2021-C-C) previously collected from diseased wild-caught eels underwent the same procedure to confirm the generalizability of growth kinetics (Supplementary Figure 2).

### 2.4 Fish for *in vivo* experiments

Approximately 375 live eels (*Anguilla rostrata*) with a mean body length of 33.8 ± 4.9 cm and weight of 55.9 ± 26.9 g (standard deviation of mean) were obtained from an eel aquaculturist in Connecticut, United States. These eels were commercially harvested primarily from Eastern Bay, a tributary of the Chesapeake Bay. Eels were transported live to VIMS and subjected to a formalin bath (at 1 mL/3.79 L for 30 min) to clear gill monogenean and other external parasites immediately upon arrival. Fish were then sorted visually for RSD, and all diseased eels (approximately 10) were euthanized. The euthanasia procedure involved immersing eels for 15 min in 1–2 L of water containing 400 mg/L of tricaine methanesulfonate (MS-222), buffered to pH 7 with 600 mg/L of sodium bicarbonate. No other clinical diseases were observed, however, to rule out the possibility of sub-clinical infections, the approximately 365 remaining eels were quarantined for 3 months prior to the experiment.

During quarantine, the eels were held in an 8,000 liter recirculating indoor fish culture system, comprised of 6 tanks and equipped with biological, UV, and mechanical filtration. The system was continuously supplied with natural saltwater (∼18 ppt salinity York River water) filtered to 3 microns through a cartridge filter chain (25, 10, 5, and 3 μM pore size) at 23°C. Eels were fed red wiggler worms (*Eisenia fetida*), purchased from a local bait shop, *ad libitum* (1–2% body weight every 3–4 days) during quarantine. To reduce stress, fish were provided with small housing units (half 6-inch PVC domes), and light was kept on a 12 h-diurnal cycle. Approximately 100 eels that either became diseased or died during quarantine were euthanized (as described above) and not used for experiments. Plausible sources of disease include holding stress and secondary infections (Mellergaard and Dalsgaard, 1987), 70% of which occurred during the first 2 weeks of quarantine and then resolved to less than 1 moribund animal every 2–3 days. Experiments were started once mortality had subsided.

### 2.5 Bacterial infection experiment

Eels were moved from the quarantine system to an aquatic pathogen BSL-II system and acclimated for 10 days prior to the start of the experiment. A total of 159 eels were randomly distributed into 6 recirculating systems, each receiving 24–28 fish. Each system consisted of three 200 liter tanks (8–9 eels per tank) connected in a closed recirculating arrangement with biological and mechanical filtration. The systems were supplied with artificial sea water made to 10 ppt salinity by adding Instant Ocean Sea Salt^®^ to UV irradiated well supplied freshwater (Figure 1).

**FIGURE 1:**
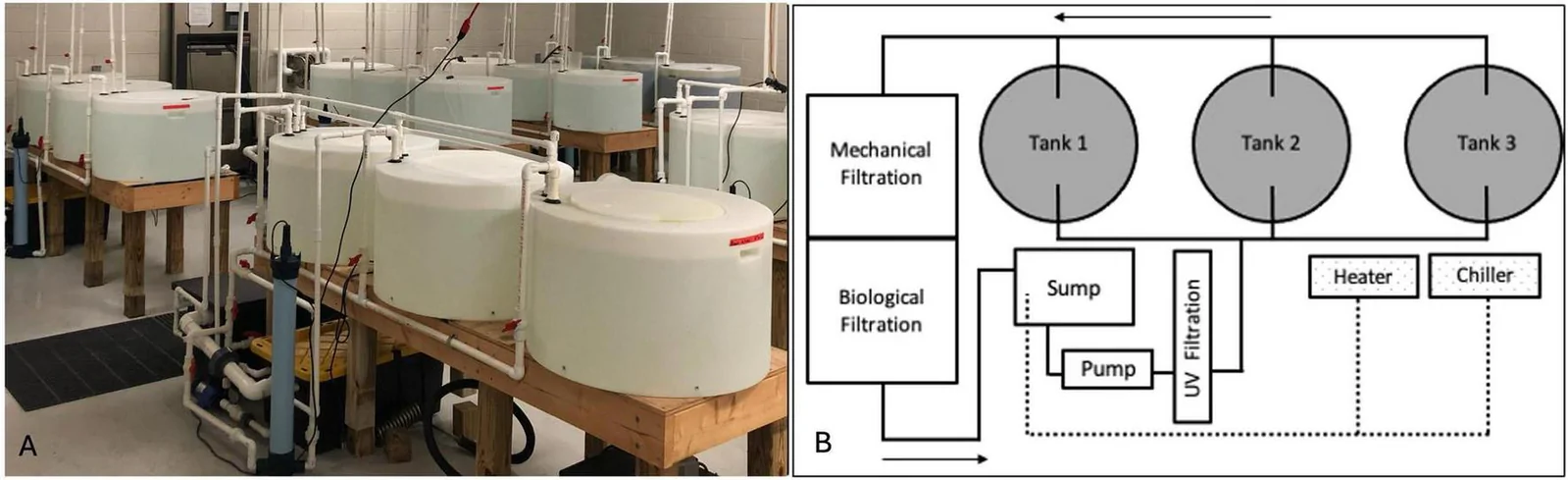
Setup of disease exposure systems in BSL-II laboratory. **(A)** Photograph of the eel housing systems used. Each system was a different treatment to avoid cross-contamination due to water recirculation within systems. **(B)** Schematic of a single system showing the three interconnected tanks, various filtration methods, and other components (modified from Gauthier et al., 2021). The UV filtration for the systems was turned off post-injections.

Each system housed one of the following treatments: (1) Pathogen (*V. vulnificus*) & low temperature (18°C), (2) No pathogen & low temperature (18°C), (3) Pathogen & optimum temperature (25°C), (4) No pathogen & optimum temperature (25°C), (5) Pathogen & high temperature (32°C), (6) No pathogen & high temperature (32°C) (Figure 2). Treatments were separated by systems to avoid cross contamination through water recirculation. Eels within a treatment were divided between the 3 tanks for each system to avoid crowding stress, which were treated as pseudo-replicates (see section 2.8). The specific temperatures were chosen to provide (1) little to no stress for eels and slow bacterial growth (18°C), (2) minimal stress for eels and moderate bacterial growth (25°C), and (3) high stress for eels and high bacterial growth (32°C), based on eel physiology (Barila and Stauffer, 1980; Tzeng et al., 1998; Jessop, 2010), and *in vitro* culture growth kinetics of *V. vulnificus* isolate AK-2020-508B. Eel holding systems were maintained at different temperature regimes using submersible heaters (TH-S 800W, Finnex) and chillers (Cyclone CY-5, Aqua Logic). Beyond temperature and pathogen treatment differences, each eel housing system was identical to each other, with the same initial water source, flow rates, filtration, and light control. No mechanical issues with any systems occurred during the experiment.

**FIGURE 2:**
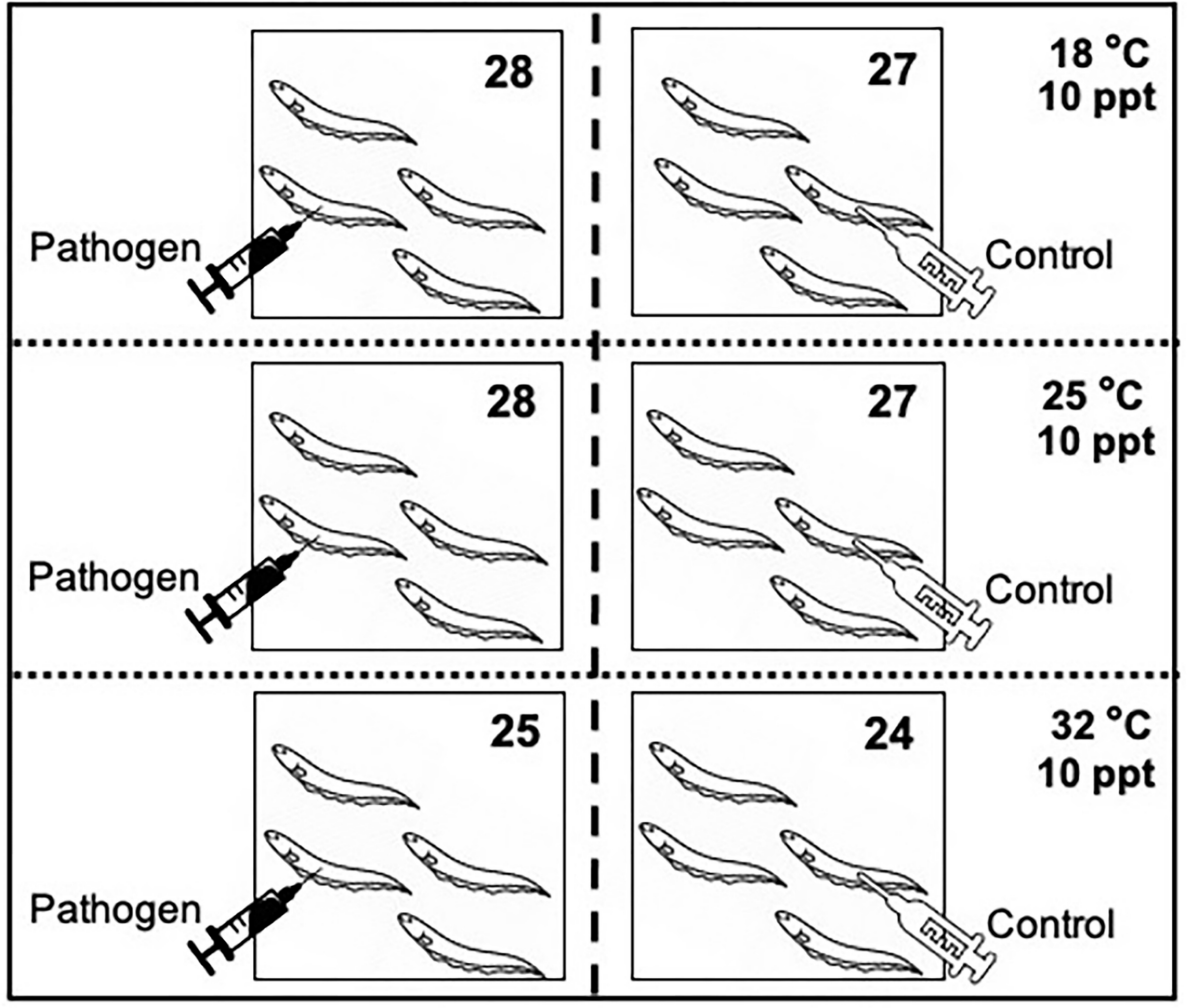
Schematic of the disease exposure experiment. Each box represents a different treatment (combination of pathogen—*V. vulnificus* or sham injection control and temperature- 18, 25, or 32°C). The number in right corner of each box is the total eels in that treatment. Filled syringes represent pathogen and empty syringes represent control. Dashed vertical line separates the treatments based on pathogen vs. control. Dotted horizontal lines separate the treatments based on temperature. Artificial seawater at a salinity of 10 ppt was used in all tanks.

Eels undergoing pathogen exposure received 100 μL of *V. vulnificus* in TSB at 7.5 × 10^4^ CFU/mL by intraperitoneal (IP) injection using a sterile 26.5G needle in a plastic syringe, with a repeat pipetter (Repeater^®^ plus, Eppendorf). Injections were administered approximately 2 cm anterior of the anus, on the left ventral side. Before injection, fish were anesthetized in an ice-bath for 5–10 min until unresponsive to stimuli. After injection, fish were allowed to recover for 5–10 min in 20-L buckets before return to the housing system. No fish had pre-existing skin lesions near the injection site. To prepare the inoculum, *V. vulnificus* isolate AK-2020-508B was revived from the −80°C glycerol stock on a TSA plate incubated at 32°C for 24 h. A single colony was then picked and inoculated in a 15 mL tube containing 5 mL TSB at 35°C for 4 h in an orbital incubator at 120 rpm. To estimate *V. vulnificus* concentration an OD_600_ reading of culture was measured to be 0.747, which corresponded to approximately 1.73 × 10^9^ CFU/mL as per the prior established OD_600_-CFU curve (Supplementary Figure 1). To prepare 50 mL of injection material at the desired dose (1 × 10^4^ − 1 × 10^5^ CFU/mL), 2.89 μL of this broth culture was added to 49.997 mL of sterile TSB. Exact titer of the injection material was confirmed later by the CFU plating method replicated twice (7.8 × 10^4^ and 7.2 × 10^4^ CFU/mL, respectively). Fish in the no-*Vibrio* control treatments received a 100 μL IP injection of the same *V. vulnificus* inoculum, after 0.22 μm syringe filtration two times. This ensured that TSB injection did not cause pathology and that residuals toxins were not produced by bacteria in culture. Sterility of the filtered TSB inoculum used in the experiment was confirmed via culture on a TSA plate (as above), which showed no colony growth.

After inoculation, the eels were maintained in the housing systems for 12 days until mortality subsided in the *V. vulnificus* injection tanks. All tanks were checked twice daily and dead or moribund eels (large lesions, swimming, or breathing difficulty) were removed from the tanks, euthanized (as described above), necropsied, and sampled for bacteriology and histology. Animals were not fed during the experiment to reduce production of biological wastes and to maintain adequate water quality (dissolved oxygen and ammonia). Temperature and salinity were measured daily.

### 2.6 Gross examination of eels and bacteriology

All fish that died during the experiment were grossly examined for clinical signs of RSD, including at the site of injection, as described by Kohli et al., 2025. During the 12-day experiment, 50 mortalities and 2 fish euthanized due to severe clinical signs (both euthanized fish from 25°C *V. vulnificus* treatment) were recorded, out of which 35 fish (33 *V. vulnificus* + 2 control inoculations) were selected for bacterial isolation from select skin lesions (if present) and livers (Table 1). The spleens of the first 12 mortalities were also sampled for bacterial isolation. Total length (cm) and weight (g) were recorded for all fish dissected. All survivors at the end of the experiment were also examined for clinical signs of RSD at the site of injection and 3 surviving fish (1 fish from each tank) were randomly sampled from each of the 6 treatments for bacteriology (Table 2).

**TABLE 1:** Number (n) of eel mortalities and downstream tissue processing across treatments.

Treatment	Mortalities (n)	Dissected (n)	Of eels dissected (n)		
			*Vibrio* isolated	Clinical signs present (at injection site)	Processed for histology
18°C control	0	0	0	0	0
18°C pathogen	10	8	8	2 (1)	2
25°C control	0	0	0	0	0
25°C pathogen	16	16	16	12 (8)	9
32°C control	4	2	1	0	0
32°C pathogen	22	9	9	1 (1)	1

A subset of eels was dissected for *V. vulnificus* re-isolation, clinical sign assessment, and histopathology (last 3 columns). Numbers in parentheses in column 5 indicate eels exhibiting clinical signs specifically at the injection site.

**TABLE 2:** Number (n) of surviving eels and downstream tissue processing across treatments.

Treatment	Survivors (n)	Dissected (n)	Of eels dissected (n)		
			*Vibrio* isolated	Clinical signs present (at injection site)	Processed for histology
18°C control	27	3	0	0	0
18°C pathogen	18	3	1	1 (0)	1
25°C control	27	3	0	1 (0)	0
25°C pathogen	12	3	1	2 (1)	1
32°C control	20	3	0	0	1
32°C pathogen	3	3	0	0	1

A subset of eels was dissected for *V. vulnificus* re-isolation, clinical sign assessment, and histopathology (last 3 columns). Numbers in parentheses in column 5 indicate eels exhibiting clinical signs specifically at the injection site.

For bacteriology, infectious material was aseptically collected from tissues and streaked on TSA plates, using methods described by Kohli et al., 2025. Culture plates were incubated at 27 °C for a maximum of 48 h, then stored at 4°C if bacterial growth occurred. Morphology was recorded for all colony types. Bacterial isolates were sub-cultured in TSB broth, at 27°C for 6–8 h in an orbital incubator at 120 rpm, and stored at −80 °C in 25% v/v glycerol for all further analyses. Molecular identification of isolated bacterial strains was performed by Sanger sequencing of the 16s ribosomal RNA (rRNA) gene using primers 5′-TCCTACGGGAGGCAGCAGT-3′; and 5′-GGACTACCAGGGTATCTAATCCTGTT-3′, under conditions previously described by Kohli et al., 2025. The identity of one bacterial isolate (AK-2021-E21C) recovered from the lesion of a fish in the 25°C *V. vulnificus* injection treatment, was also confirmed using whole genome sequencing (using methods described in section 2.2).

### 2.7 Histology

Whole transverse sections (∼1.0 cm thick) of eels through the site of injection, and skin lesions when present at the site of injection, containing the visceral organs were preserved in Z-fix (zinc-formalin; Anatech) for at least 48 h. Routine methods for paraffin histology were used to process fish tissues for histopathological examinations (Prophet et al., 1994). Briefly, tissue blocks were rinsed overnight in running water to remove fixatives, decalcified overnight using an equal part solution of sodium citrate and formic acid, rinsed overnight in running tap water, trimmed with a single-edge razor blade, and placed in labeled tissue cassettes. Tissues were then stored in 70% ethanol until paraffin embedment, sectioning, and Hematoxylin and Eosin (H&E) staining of tissue sections. Tissue processing was carried out in an automatic tissue processor (Shannon Excelsior ES Processor, ThermoFisher) and involved dehydration in a graded ethanol series, clearing in an intermediate solution (Xylene), and infiltration in paraffin wax. Tissue blocks were then embedded in paraffin on a tissue embedding center (Histostar Embedding Center, ThermoFisher), and sectioned at 5 μm on a rotary microtome (Titan 5000 Rotary Microtome, Tanner). Sections were placed on glass slides and stained with H&E in an automatic slide stainer (Varistain Gemini ES Stainer, ThermoFisher). Stained slides were cover-slipped and oven-dried. Evaluation and photography of histologic sections was done using a photomicroscope (BX-50, Olympus). The number of fish processed for histology from each treatment is given in Tables 1, 2.

### 2.8 Statistical analyses

All analyses were performed in R version 4.4.2 (R Core Team, 2024) using the RStudio interface (Posit Team, 2025). Survival data was visualized using Kaplan-Meier curves (Therneau, 2021). The survival data was then analyzed using generalized linear mixed effects models (GLMM), with a binomial error distribution (i.e., logistic regression), by implementing the “glmer” function from the lme4 package (Bates et al., 2015). The response variable was survival status (alive = 0, dead = 1) of each eel at the end of the experiment (day 12), with fixed factors pathogen exposure status (categorical; control vs. *V. vulnificus*) and temperature (categorical; 18, 25, or 32 °C). Because each treatment was housed within a single experimental system containing triplicate tanks, to account for nested variance, tank was included as a random effect in the models. Initially, full models with all factors and interaction terms were explored but failed to converge. This was likely because zero mortality occurred in two out of three of the no-*Vibrio* control treatment groups. To remedy this, the analysis was broken into two parts. Firstly, the effect of overall pathogen exposure was evaluated on all data, averaging across temperatures (only the fixed factor “pathogen exposure status” was included in the model). Secondly the effect of temperature was evaluated, by only including data for fish exposed to *V. vulnificus* (only the fixed factor “temperature” was included in the model). Baselines in the analyses were set to the no-*Vibrio* control and 18°C treatments. AIC values were compared to null models to confirm best fit. Proportional hazard models were also explored (Cox—coxme package and Weibull—parfm package) but failed to converge due to violation of the proportional hazards assumption (Munda et al., 2012; Therneau, 2020). Underlying assumptions for the models were tested using diagnostic plots. Co-efficient 95% confidence intervals (CI) were calculated with the equation: *e*^(coefficient ± 1.96*SE)^.

## 3 Results

### 3.1 *In vitro* growth kinetics of *V. vulnificus*

The growth of *V. vulnificus* isolate AK-2020-508B in liquid culture media was influenced by temperature and salinity (Figure 3). Over the course of 7 h, the bacteria grew most rapidly at 35°C, followed by 27°C, and showed minimal growth at 20°C. Cultures reached the exponential growth phase by 3–4 h at 35°C and 5–6 h at 27°C, with growth entering the logistic phase at 5–6 h for only the highest temperature. Growth at 20 and 10 ppt salinity was comparable when incubated at the same temperature. However, no growth was observed at 0 ppt salinity regardless of the temperature. Similar growth kinetics were observed for *V. vulnificus* isolates AK-2020-515C, AK-2021-C-C (Supplementary Figure 2).

**FIGURE 3:**
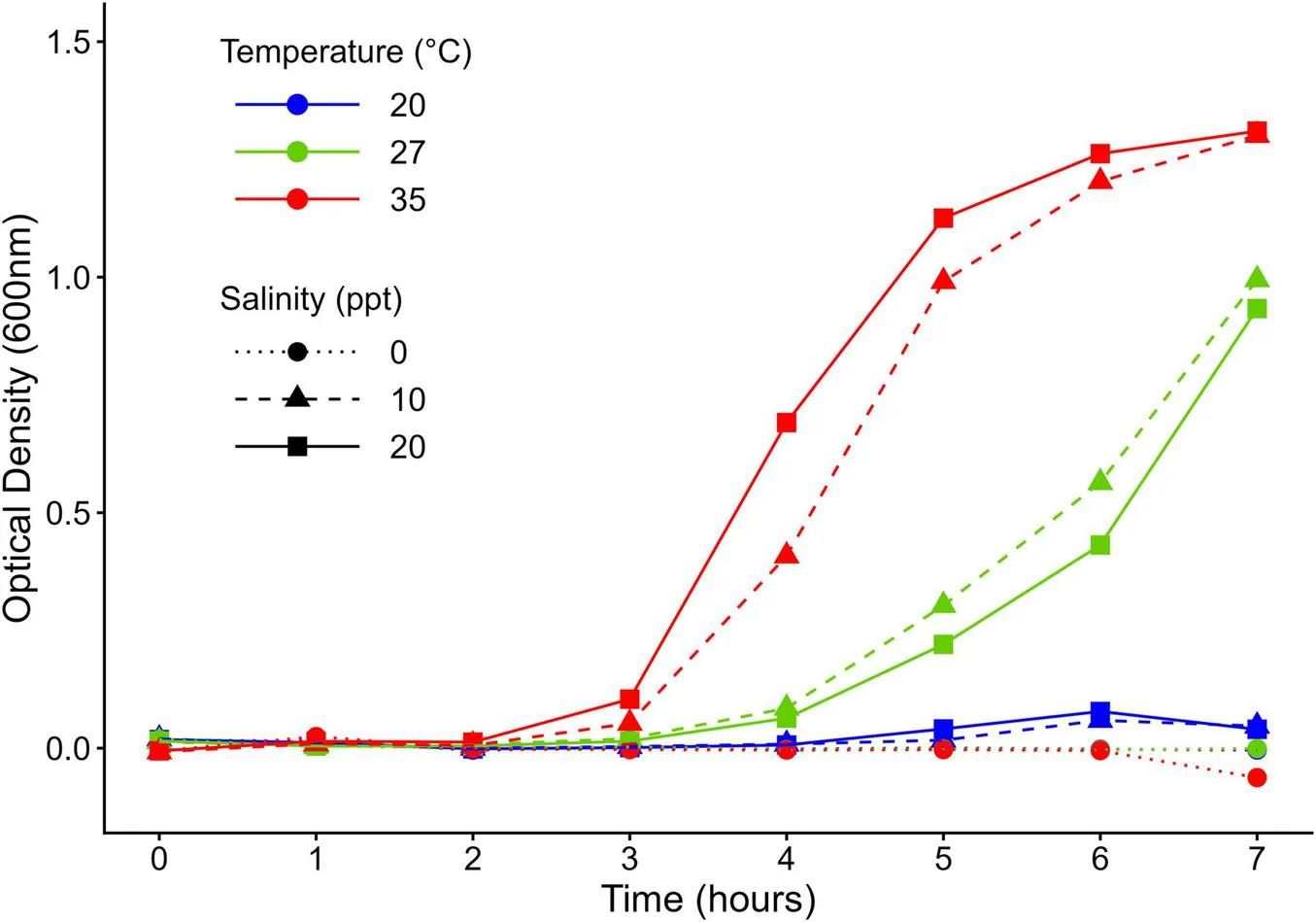
Growth kinetics of *V. vulnificus* isolate AK-2020-508B *in vitro*. The bacteria were cultured in Tryptic soy broth (TSB) at three different temperatures: 20°C (blue), 27°C (green), and 35°C (red), and three different salinities: 0 ppt (dotted line, circle), 10 ppt (dashed line, triangle) and 20 ppt (solid line, square). Growth of bacteria was measured hourly for 7 hours by optical density at 600 nm. Similar growth kinetics were observed for two other isolates tested (Supplementary Figure 2).

### 3.2 Eel survival

The survival of eels in the experiment (Figure 4) was heavily influenced by pathogen injection (ΔAIC null model = 17.6, Supplementary Table 1) and temperature (ΔAIC null model = 9.4, Supplementary Table 2). The odds of mortality was 92 (95% CI: 8.6–984) times higher for *V. vulnificus* injected versus no-*Vibrio* injected control eels (Supplementary Table 1, coefficient *p* < 0.001), when averaged across temperatures. Virtually no fish died in the no-*Vibrio* injection group, except for four fish (16.7%) in the 32°C control treatment (Table 1). When focusing on just those eels injected with *V. vulnificus*, the odds of death at 32°C was 13.2 (95% CI: 3.2–55.2) times greater than at 18°C (Supplementary Table 2, coefficient *p* < 0.001). A suggestive trend of higher odds of eels dying in the presence of *V. vulnificus* at 25°C compared to 18°C was also observed but not statistically significant (Supplementary Table 2, coefficient *p* = 0.11, 95% CI odds: 0.82–7.09).

**FIGURE 4:**
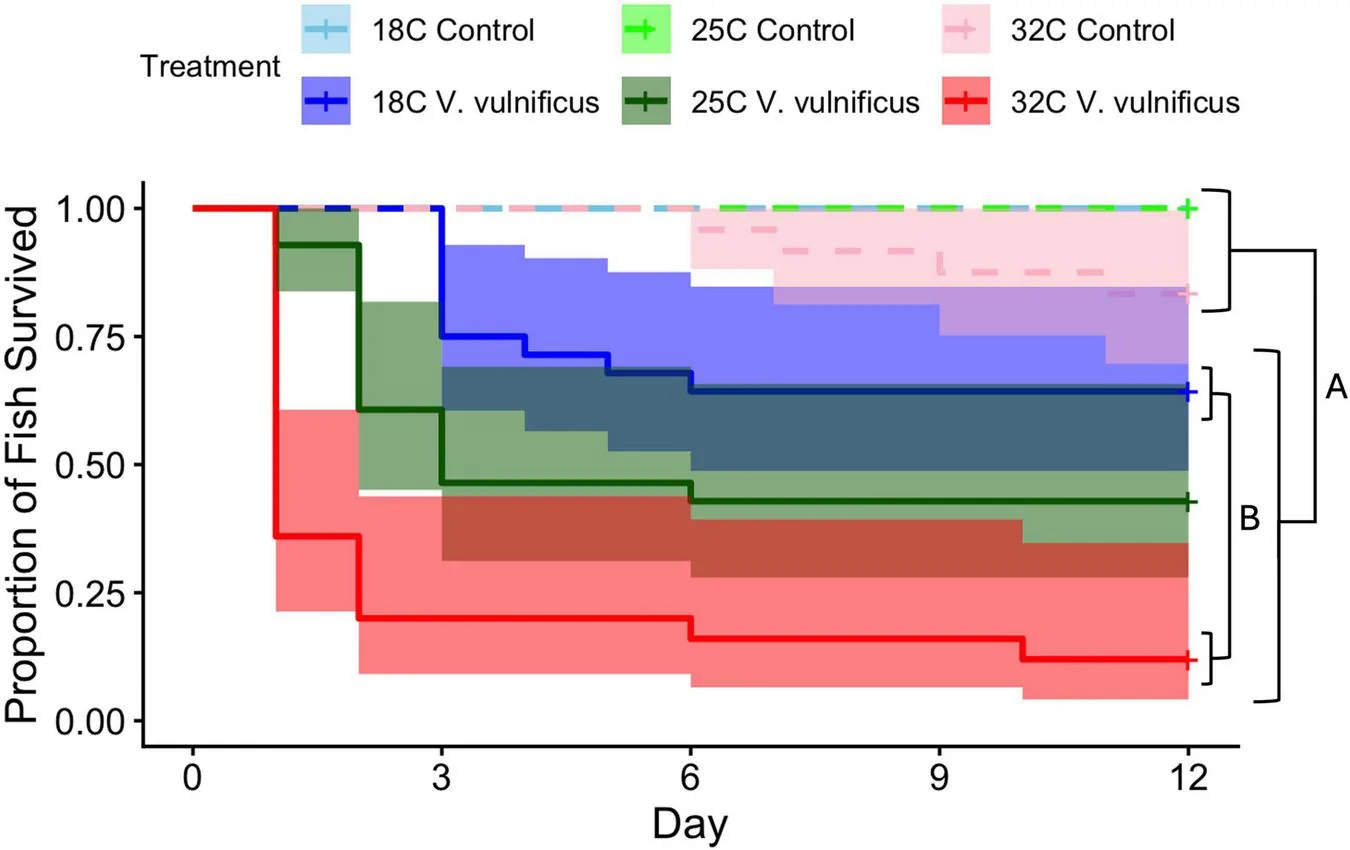
Eel survival during *in vivo* experiment. Lines show Kaplan-Meier survival curves of eels for each temperature (blue = 18°C, green = 25°C, and red = 32°C) and pathogen treatment (*V. vulnificus* injection = solid line, darker colors; sham injection control = dashed line, lighter colors), with 95% confidence intervals (shading). Survival curves for 18°C and 25°C sham injection controls are superimposed with zero mortality in both treatments. Brackets show statistical difference between: **(A)** no-*Vibrio* control versus *V. vulnificus* injection treatments, averaged across temperature (*p* < 0.001, Supplementary Table 1), **(B)** 18°C versus 32°C for *V. vulnificus* injection treatments (*p* < 0.001, Supplementary Table 2), from logistic regression analysis.

The kinetics of eel mortality after *V. vulnificus* injection also qualitatively increased with temperature (Figure 4). For eels held at 18°C, mortality began and peaked on day 3, then ceased on day 6. In contrast, eel mortality at 25°C peaked on day 2 and plateaued by day 3. Whereas eels held at 32°C started to die and reached peak mortality by day 1, with over 80% of deaths observed within the first 2 days of the experiment. No mortalities were recorded in the *V. vulnificus* injection treatments after day 6, except one fish at 32°C on day 10. The low-level mortality observed in the no-*Vibrio* injection control groups at 32°C began on day 6, then remained low level and evenly spaced till ceasing on day 10.

### 3.3 Clinical signs and pathology

External clinical signs of RSD after injection with *V. vulnificus* included hemorrhage, dermal erosion, and superficial to deep lesions (as described in Kohli et al., 2025), typically near the injection site. Clinical signs first appeared within 24 h post-infection, reaching peak prevalence by day 2–3. Lesions were present at or near the injection site on 10 of the 33 dead fish sampled from the *V. vulnificus* injection treatments (Table 1). External clinical signs across surviving and dead eels (e.g., lesions) that were dissected were most frequently observed in the 25°C *V. vulnificus* treatment (14/19 eels), with similar low prevalence for the 32°C (1/12) and 18°C (3/11) pathogen treatments (adding across Tables 1, 2). No clinical signs consistent with RSD were present in eels in the no-*Vibrio* injected control treatments. One surviving eel in the 25°C no-*Vibrio* control treatment, along with one surviving eel from both the 18 and 25°C *V. vulnificus* treatments presented with small, non-descript, caudal peduncle lesions not characteristic of RSD and far from the site of injection. Except for one eel in the 25°C *V. vulnificus* injection treatment, none of the remaining survivors across any treatment exhibited clinical signs consistent with RSD at the site of injection.

Gross pathology and histological examination (Tables 1, 2) provided a clear pattern of disease progression. In fish without external clinical signs of RSD (Figure 5A), histologically, the skin exhibited an intact multi-layered outer epidermis, and a healthy underlying dermis and hypodermis (Figure 5B). In fish with external signs of RSD, the most common presentation of an early lesion (Figure 5C) was focal to multi-focal swelling and hyperemia (extreme in some cases), especially around the injection site and anus. These early lesions demonstrated loss of scales, partial loss of the epidermis, and edema of the dermis and hypodermis (Figure 5D). In smaller lesions, the dermis was mostly intact. As severity progressed, necrotic skin tissue became more detached from the body, in some cases with only a small connection at the edge of the lesion. Gross swelling around the site of injection, due to fluid accumulation, was also seen in some fish. In the most advanced lesions (Figure 5E), all layers of the skin were eroded exposing the underlying musculature (Figure 5F), which differed considerably from the shallow early lesions that penetrated only to the dermis (Figure 5B).

**FIGURE 5:**
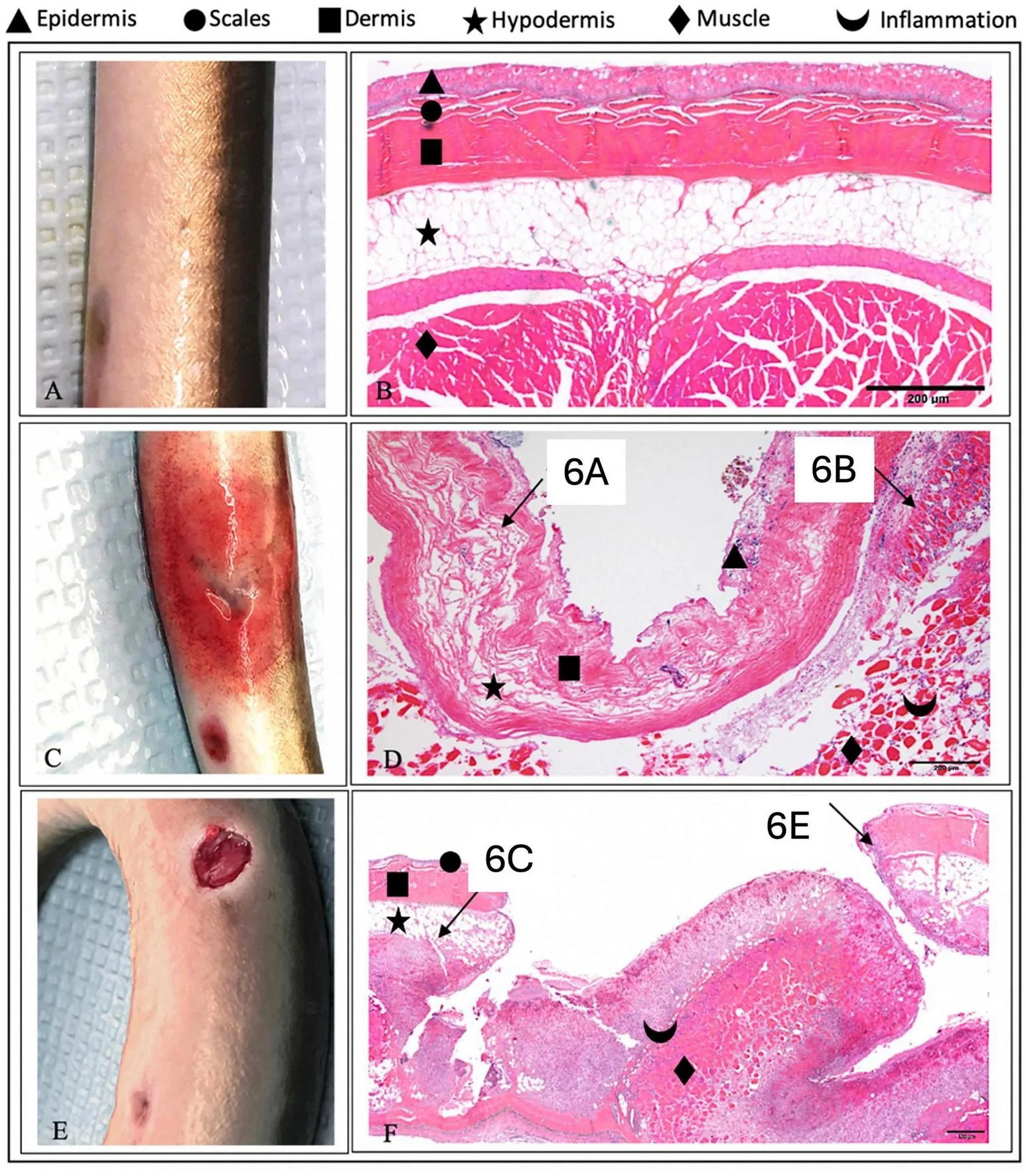
Gross pathology and corresponding histological sections (H&E) of dermal ulcers. Clinical signs during experiment were indicative of infection with *V. vulnificus* and resulting red sore disease in American eels. **(A)** Healthy injection site and anus. **(B)** Healthy cross section of body showing intact dermal layers and muscle. **(C)** Early lesion characterized by extreme hyperemia and skin erosion at site of injection, also a hyperemic anus is seen. **(D)** Loss of scales, erosion of epidermis, edema of dermis, hypodermis, and muscle with inflammation. **(E)** An advanced lesion at the site of injection. **(F)** Complete loss of dermis and intense inflammation of muscle seen in an advanced lesion. Scale bars = **(B,D)** 200 μm, **(F)** 500 μm. Arrows show areas magnified in Figure 6. Triangle = epidermis, closed circle = scales, square = dermis, star = hypodermis, diamond = muscle, crescent = inflammation.

The host inflammation response also progressed with lesion severity. Inflammatory cells were absent at the site of injection for eels without lesions or other clinical signs (Figure 5B). For early to mild lesions with edema (Figure 6A), a small number of mononuclear cells were present in the epidermis at the site of injection (Figure 6B), corresponding with bacterial colonies along the muscle planes (Figure 6B). As lesions advanced, inflammation extended deeper into the hypodermis and muscle (Figure 6C), which was accompanied by focal myonecrosis (Figure 6D). A pronounced mononuclear phagocytic response also became apparent within the epidermis and dermis (Figure 6E). At the margin of eroded skin, epidermal cell proliferation was observed (Figure 6E), suggesting an attempt by the host response to close the opening. In all fish, regardless of how advanced their lesions were, inflammation and necrosis in the liver and spleen were mostly mild. However, bacteria were often observed in the blood vessels in these organs (Figure 6F). Bacterial colonies were also observed in other internal organs such as the swim bladder and ovaries. Staining morphology and coloration indicated that all bacteria observed were Gram negative.

**FIGURE 6:**
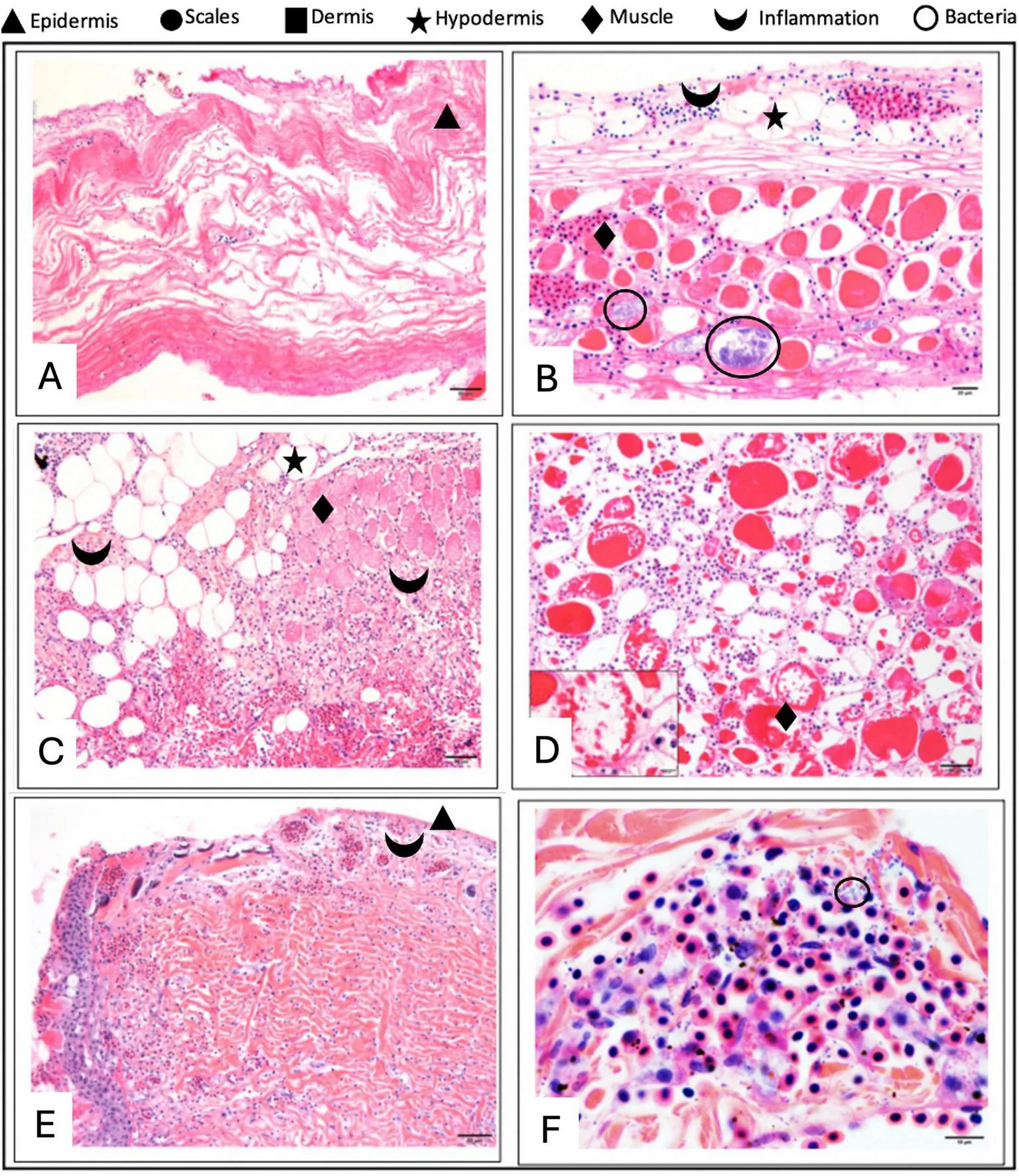
Inflammatory response associated with red sore disease in various tissues. Panels show representative histological (H&E) sections of eels experimentally infected with *V. vulnificus* showing: **(A)** Edema of dermis associated with an early lesion. **(B)** Inflammation and bacterial colonies seen in hypodermis and muscle in an early lesion. **(C)** Extreme inflammation seen in hypodermis and muscle in an advanced lesion. **(D)** Focal myofiber necrosis (shown in inset) seen in deeper muscles of a lesion. **(E)** Inflammation and regenerating epidermis seen at the edge of an advanced lesion. **(F)** Bacteria seen in a blood vessel in the liver of an eel with a systemic bacterial infection. Scale bars = **(A,C,D,E)** 50 μm, **(B)** 20 μm, **(F)** 10 μm. Triangle = epidermis, closed circle = scales, square = dermis, star = hypodermis, diamond = muscle, crescent = inflammation, open circle = bacteria.

### 3.4 Bacteriology and sequence identification

The consensus 16S rRNA gene sequence for the *V. vulnificus* isolate (AK-2020-508B) used for experimental infections in this study was 100% identical to a large number of GenBank deposited *V. vulnificus* sequences. The *V. vulnificus* identity of the isolate was further confirmed through whole genome sequencing. Targeted exploration of the genome indicated that the *gyrB* and *lysB* gene sequences had a 100% match to *V. vulnificus* CECT4999, originally isolated from *Anguilla anguilla* in Spain (Lee et al., 2008).

Bacteria were recovered from the lesions at the injection sites, liver, and spleens of all the 33 eel mortalities that were sampled from the *V. vulnificus* injection treatments. These bacterial isolates grew as pure cultures of opaque buff-colored colonies approximately 2 mm in diameter and were identified to be *Vibrio* spp. through 16S rRNA Sanger sequencing (Tables 1, 2). The whole genome for one isolate (AK-2021-E21C), recovered from the lesion of a fish in the 25°C pathogen treatment, was sequenced and further identified to be *V. vulnificus*. This matched with the whole genome identification of the original isolate AK-2020-508B used for the experimental infections.

Two eels that died in the 32°C no-*Vibrio* control group were necropsied, and the liver samples tested positive for bacteria via culture for one eel but not the other (Table 1). Of the nine-surviving eels sampled from the *V. vulnificus* injection treatments, bacteria were successfully cultured twice—once from a caudal peduncle lesion on an eel in the 18°C treatment, and once from the liver of an eel in the 25°C treatment that presented a deep lesion at the site of injection. All three isolates (1 dead control + 2 surviving pathogen treatments) were identified as *Vibrio* spp. through 16S rRNA sequencing. Another survivor sampled from the 25°C *V. vulnificus* injection treatment had a caudal peduncle lesion, but no bacteria were recovered. Nine no-pathogen control eel survivors were also sampled, including one presenting with caudal peduncle lesions, but no bacteria were recovered from the internal organs or lesion in these fish (Table 2).

## 4 Discussion

Red sore disease (RSD) has been reported as an emerging infectious disease in aquaculture and the wild for American eels in the Chesapeake Bay region, of the United States (Kohli et al., 2025). It is common for various bacterial species or other infectious and non-infectious agents to produce the generalized pathology associated with RSD in fish (Noga, 2000; Law, 2001). Earlier reports of the disease in American eels from North Carolina and South Carolina in the 1980s identified *aeromonads* to be the causative pathogen. At that time, *vibrios* were rarely isolated and never implicated as an etiological agent for RSD in American eels (Hayasaka and Sullivan, 1981; Inman and Bland, 1981; Davis and Hayasaka, 1983; Noga and Berkhoff, 1990). However, in Kohli et al., 2025, we reported *Vibrio* spp. infections to be highly associated with the disease, especially when septicemia was present, but their role as causative agents in the recent RSD outbreak in the Chesapeake Bay has not been previously experimentally verified.

Here, we obtained a *V. vulnificus* isolate from a wild-caught eel showing clinical signs of RSD, harvested in 2020 from the Chesapeake Bay. We then experimentally infected clinically healthy American eels to confirm the pathogenicity of *V. vulnificus* and fulfill Koch’s postulates (Evans, 1976). We (1) observed clinical signs and mortality kinetics in *V. vulnificus* injected eels, consistent with those reported from naturally infected eels (Kohli et al., 2025). We then (2) successfully re-isolated the bacteria as pure cultures from lesions at the site of injection from experimentally infected eels. We also (3) observed no RSD clinical signs or isolation of bacteria at the site of injection in no-*Vibrio* control groups, despite low level mortality under the highest temperature stress treatment. Genetic sequencing, in addition to the consistent gross and histopathology observed by us and others (Tison et al., 1982; Biosca et al., 1991; Fouz et al., 2006; Haenen et al., 2014; Xu et al., 2024), strongly support that all isolates recovered from experimentally infected eels were *V. vulnificus*. To the best of our knowledge, this is the first report fulfilling Koch’s postulates to confirm *V. vulnificus* isolates from North America as an etiological agent of RSD in American eels. These findings are consistent with those from Europe and Asia where an ulcerative condition similar to RSD has been described from eels (*A. anguilla* and *A. japonica*, respectively) and attributed to *V. vulnificus* biotype 2 (Tison et al., 1982; Biosca et al., 1991; Fouz et al., 2006; Haenen et al., 2014). The bacteria used for the exposure study here also matched closely with a biotype 2/Serovar E isolate, *V. vulnificus* CECT4999, recovered from kidneys of a diseased eel in Spain, based on whole genome sequencing (Valiente et al., 2008; Roig et al., 2010).

During the experiment, many eels developed clinical signs typical of RSD including hyperemia and hemorrhage, dermal erosion and necrosis, as well as superficial to deep necrotic ulcers. Some animals, although septicemic, died without presentation of clinical signs. In fact, clinical signs amongst *V. vulnificus* injected eels were most common at the intermediate holding temperature (25°C) but rarely observed at the highest temperature (32°C). This may indicate that eels under high temperature died too rapidly to allow clinical disease progression, whereas under lower temperatures at their thermal optimum the eels were able to manage infection. The inconsistent presentation of external clinical signs after IP delivery of the bacteria may support the speculation of inside-out progression of lesions in the case of experimental infection.

Common histological findings due to *V. vulnificus* injection included loss of the epidermis, as well as edema of the dermis and hypodermis due to a mononuclear inflammatory infiltrate. Isolation of bacteria from the internal organs of the fish confirmed a systemic *V. vulnificus* infection as the cause of death in most fish, further supporting our Koch’s postulates conclusions. It appears that some fish were also successful at recovering from the infection, given that mortality never reached 100% in any of the *V. vulnificus* injection groups. Indeed, in one of the survivors from the pathogen treatment group with a deep lesion at the site of injection, bacteria were isolated from the liver but not the lesion, indicating local control of infection. Regenerating epidermis was also seen in a histological section of this lesion, suggesting a healing attempt to wall off the open lesion. The mechanism behind the observed variation in individual eel susceptibility to *V. vulnificus* infections is unknown. Some fish may have had acquired immunity from a previous infection or exhibit a greater resistance due to genetic differences. Since the history of these eels is uncertain, more investigation on these factors is needed, however, the fish were obtained from environments where *V. vulnificus* is known to circulate. Here the goal of histology was to confirm the presence or absence of disease. Future expanded quantitative histological as well as molecular studies might provide additional insights into the cellular and immune progression of clinical disease and recovery. A recent study indicated that pathology due to *V. vulnificus* infection in American eels is likely attributable to immunosuppression (Xu et al., 2024), although temperature effects were not investigated.

We attempted to exclude all eels from this study with prior subclinical *V. vulnificus* or other pathogen infections. We note that these were wild caught animals and some included did have small lesions on the caudal peduncle. We do not believe these common shallow caudal peduncle ulcers represent clinical signs of RSD, given that they rarely harbored bacteria, as determined through our culturing efforts. However, we cannot completely rule out the possibility that some eels carried *V. vulnificus* before the start of the experiment, given its ubiquitous presence in the environment. Formalin treatment and subsequent quarantine likely removed pathogens from the vast majority of animals. Although mortality in the quarantine was observed, this is not unheard of in wild caught animals, even after antibiotic treatment and quarantine (Mellergaard and Dalsgaard, 1987; Barreiro et al., 2017; Hadfield, 2021). Holding stress may have contributed to mortality during quarantine, which we attempted to minimize by maintaining high water quality and providing hiding structures for eels. In our study, mortality had largely subsided before the experiment began, indicating any potential underlying infection had cleared. This is supported by our finding that *V. vulnificus* was only isolated from one no-*Vibrio* treatment control fish that died during the experiment. Likewise, bacteria were not isolated from any of the survivors from the control treatments after the experiment. Our results indicate that if background low-grade infections were present, they were rare, and only progressed to disease in the presence of extreme temperature, given that mortality was not observed in no-pathogen injection controls at 18 and 25°C. In the one treatment where disease did occur in control eels, maintained at 32°C, it progressed much slower and reached only 15% compared to the greater than 40% mortality reached in *V. vulnificus* injected eels. This indicates that temperature stress rather than *V. vulnificus* was the driver of mortality in these eels. Furthermore, when lesions on the caudal peduncle were present, bacteria were isolated from fish in the *V. vulnificus* exposure treatment but not from the control treatment. Although not directly tested, this may suggest that these lesions potentially resulted from injury and acted as a portal of entry where water was the mode of transmission for the bacteria shed from injected fish, which has been reported by others (Amaro et al., 1995; Marco-Noales et al., 2001; Fouz et al., 2010). Caudal peduncle lesions are frequently observed in eel aquaculture due to injury during harvest or housing and could be a site of bacterial entry under stressful conditions. Regardless of any low-level underlying infections the eels may have had, *V. vulnificus* injection rapidly and drastically increased the odds of mortality and disease, supporting our conclusion that experimental infection was the causative agent of the observed RSD.

Another goal of our study was to determine how temperature might interact with *V. vulnificus* exposure to influence the severity of RSD. In our experiment, an overall mortality of 59.25% was recorded for the fish in the *V. vulnificus* injection treatments. On the contrary, an overall mortality of only 5% was recorded for the fish in the no-*Vibrio* control treatments, with all mortality occurring only at the highest temperature of 32°C. These findings indicate that elevated temperature on its own may cause some mortality to animals, suggesting its role as a stressor, but the effect is much greater when *V. vulnificus* infections are present. The exact mechanisms for this interactive temperature effect on the progression of *V. vulnificus* infections in American eels is uncertain. Our *in vitro* experiment showed that the growth of the bacteria was more rapid at 32 than 18°C. This indicates increased *V. vulnificus* proliferation at higher temperatures may be elevating mortality. Some indirect effects of eel stress might also be at play. American eels are known to tolerate a wide range of temperatures (Van Den Avyle, 1984; Shepard, 2015). However, stress, through the action of corticosteroids, can compromise the natural defenses of fish against invading pathogens by affecting lymphocyte count and antibody-production capacity (Barton and Iwama, 1991). Thus, a stress cascade induced by high temperature could have made the eels more susceptible to *V. vulnificus* infection and reduced immune clearance. We note that this stress response was not directly measured in this study, and quantification of temperature effects on hormone and immune activity in association with RSD warrants further investigation. Another interesting finding of our *in vitro* experiment was that the growth of *V. vulnificus* was hampered in freshwater. This finding was consistent with observations by Marco-Noales et al., 1999 who found that survival of *V. vulnificus* isolated from diseased European eels was only possible at low salinities if temperature was high.

It is plausible that the intraperitoneal injection challenge used in this study bypassed some aspects of the natural infection process, such as mucosal immunity and epithelial barriers. Previous studies have shown that route of *V. vulnificus* infection can impact disease severity in European eels (*Anguilla anguilla*), with waterborne immersion challenges still inducing high levels of mortality, but slower disease progression than injection or direct fish-to-fish contact (Amaro et al., 1995; Marco-Noales et al., 2001). To our knowledge, such route of infection comparison studies have not been conducted in American eels. Given prior evidence of injury as a transmission route of RSD in American eels in Aquaculture (Kohli et al., 2025), IP injection was used as the most consistent and controlled way to simulate entry when the dermis is bypassed, such as through injury. This approach also allowed us to ensure all animals received the same pathogen dose for experimental standardization and reproducibility purposes. Given that clinical signs consistent with red sore disease were only observed in *V. vulnificus* and not sham injected animals, there was no indication that any aspect of the injection process alone was pathogenic. The impact of alternate routes of infection on disease progression in American eels, through waterborne exposure, direct contact, or even ingestion in feed, warrants further investigation, given that RSD has also been observed in individuals without overt injuries (Kohli et al., 2025). This may facilitate inferences of field processes, where a myriad of host, pathogen, and environmental factors are likely to influence transmission. Disentangling these interactions was beyond the scope of our study. However, our results provide strong support for increasing disease severity with higher temperatures, once infection was established.

The effects of temperature on infectious diseases in finfish are common to various systems and known to have population level disease impacts in light of climate change (Marcogliese, 2008; Mohanty et al., 2010; Chiaramonte et al., 2016). Temperatures above 30°C have been recorded both in American eel aquaculture operations (Kohli et al., 2025) and regional wild eel habitat (Chesapeake Bay National Estuarine Research Reserve in Virginia, Virginia Institute of Marine Science [CBNERR-VAVIMS], 2022). It is predicted that temperature increases due to climate change will result in higher *V. vulnificus* abundance in the Chesapeake Bay, due to more favorable bacterial growth conditions (Jacobs et al., 2010; Muhling et al., 2017). Although speculative, this may be a driver behind field observations indicating that RSD due to *Vibrio* spp. has become more prevalent in the Chesapeake Bay. Climatic changes over the past few decades might also explain why studies of RSD in American eels from North and South Carolina in the 1980s implicated aeromonads rather than vibrios, given that aeromonads tend to favor lower temperatures (Kohli et al., 2025). Inferring the impact of climate change on the causes, prevalence, and severity of RSD in American eels will require additional research.

*V. vulnificus* (of Biotype 2 identified here) can also infect humans (Amaro and Biosca, 1996; Heng et al., 2017), posing a dual threat due to its high pathogenicity for eels and its increased human health risks. In addition to gastrointestinal foodborne illness, of particular concern is severe sepsis and skin and soft-tissue infection via dermal exposures through wounds for recreational swimmers and fish farmers (Hoge et al., 1989; Horseman and Surani, 2011; Shaw et al., 2015). Over the past few decades, *V. vulnificus* wound infections in humans have shown an eightfold increase alongside a rapid northward range expansion of the pathogen in the Eastern USA (Archer et al., 2023). Nationally, Hast et al., 2026 report that the overall *V. vulnificus* cases grew by 70% over the last two decades. Furthermore, non-foodborne environmental and wound exposures have emerged as the dominant infection pathway, accounting for 54% of all documented cases, which constitutes roughly 2.5 times more infections that traditional foodborne routes (Hast et al., 2026). Ultimately, these trends underscore an escalating public health burden from *V. vulnificus* infections in the United States that will likely only intensify, given that climate models predict the pathogen will establish a presence in every Eastern US state by 2100 (Archer et al., 2023). Consequently, mitigating these rising zoonotic exposure risks is a critical area of future research to safeguard both coastal communities and the aquaculture industry.

The results provided here, demonstrating an effect of temperature on *V. vulnificus* infections in eels, also provide insights for disease mitigation in American eel aquaculture as well as risk assessment in the wild. Given that American eels are tolerant of a wide range of salinities and temperatures (Van Den Avyle, 1984; Shepard, 2015), practicing American eel aquaculture in controlled low temperature freshwater conditions may help mitigate disease-associated mortality due to RSD caused by *V. vulnificus*, as has been suggested for European eels (Amaro et al., 1995; Fouz et al., 2006). Furthermore, regions prone to high temperatures, particularly because of climate change, may experience increased *V. vulnificus* prevalence and RSD severity in American eels in the wild. The role of environmental stress on the prevalence of disease in American eels and other species prone to *Vibrio* spp. infections, is an important area of future research.

## Data Availability

The data presented in the study are deposited in the Figshare repository, accession number https://doi.org/10.6084/m9.figshare.33352107.
