# Supplementary material for: Elevated temperature exacerbates red sore disease caused by Vibrio vulnificus in American eels (Anguilla rostrata)
Source: Front Microbiol. 2026 Oct 5;17:1911288. doi: 10.3389/fmicb.2026.1911288 (PMC13677888; doi:10.3389/fmicb.2026.1911288)
Supplement: Supplementary file 1 [file Supplementary_file_1.pdf]

Supplementary Information

Table S1: Parameter estimates generalized linear mixed effects model (GLMM) for pathogen effect on eel survival. All data was included, with effect averaged across temperatures. Response variable = fish survival status (alive = 0, dead = 1), at the end of the 12-day experiment. Predictor = pathogen exposure status (control vs. *V. vulnificus*). Random factor = 1|tank to account for tank effects. Units are log-hazard (logit) of death; e^{Estimate} = back transformed odds. Baseline = control.

Factor	Estimate	Standard Error	e^{Estimate}	z-value	p-value
Intercept	-4.00	1.05	NA	-3.80	0.00015
V. vulnificus	4.52	1.21	91.74	3.74	0.00018

Model: $\ln\left(\frac{p_{ij}}{1-p_{ij}}\right) = \beta_0 + \beta_1 Pathogen_i + \mu_j, y_{ij} \sim Binomial(n_{ij}, p_{ij})$, where i = Pathogen exposure status (2 levels), j = tank (18 levels)
AIC: 137.2
Residual degrees of freedom: 156
AIC null model: 154.8 (ΔAIC = 17.6)

Table S2: Parameter estimates generalized linear mixed effects model (GLMM) for temperature effect on eel survival. Only data for eels exposed to pathogen were included. Response variable = fish survival status (alive = 0, dead = 1), at the end of the 12-day experiment. Predictor = temperature (categorical, 18, 25, 32ºC). Random factor = 1|tank to account for tank effects. Units are log-hazard (logit) of death; e^{Estimate} = back transformed odds. Baseline = 18 ºC.

Factor	Estimate	Standard Error	e^{Estimate}	z-value	p-value
Intercept	-0.59	0.39	NA	-1.49	0.14
Temperature = 25	0.88	0.55	2.41	1.6	0.11
Temperature = 32	2.58	0.73	13.20	3.53	0.00042

Model: $\ln\left(\frac{p_{ij}}{1-p_{ij}}\right) = \beta_0 + \beta_1 temperature_i + \mu_j, y_{ij} \sim Binomial(n_{ij}, p_{ij})$, where i = temperature (3 levels), j = tank (9 levels)
AIC: 101.1
Residual degrees of freedom: 77
AIC null model: 110.5 (ΔAIC = 9.4)

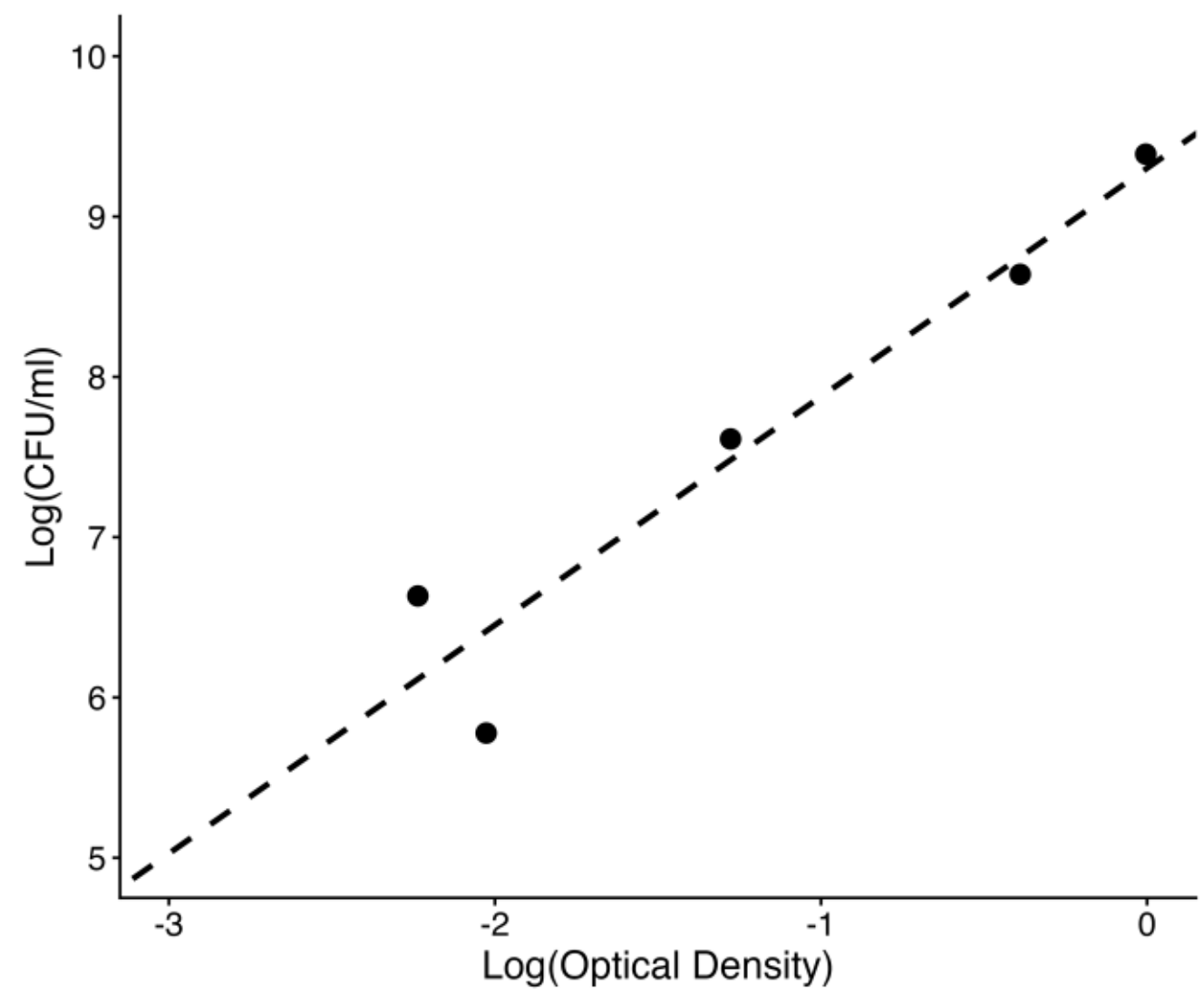

Fig. S1. Relation between log(OD_{600}) and log(CFU/ml) established from a broth culture of *V. vulnificus* (AK-2020-508B) grown at 35°C in 10 ppt salinity. Trendline shows best fit curve ($y = 1.425x + 9.302$, R^2=0.90, $p = 0.011$).

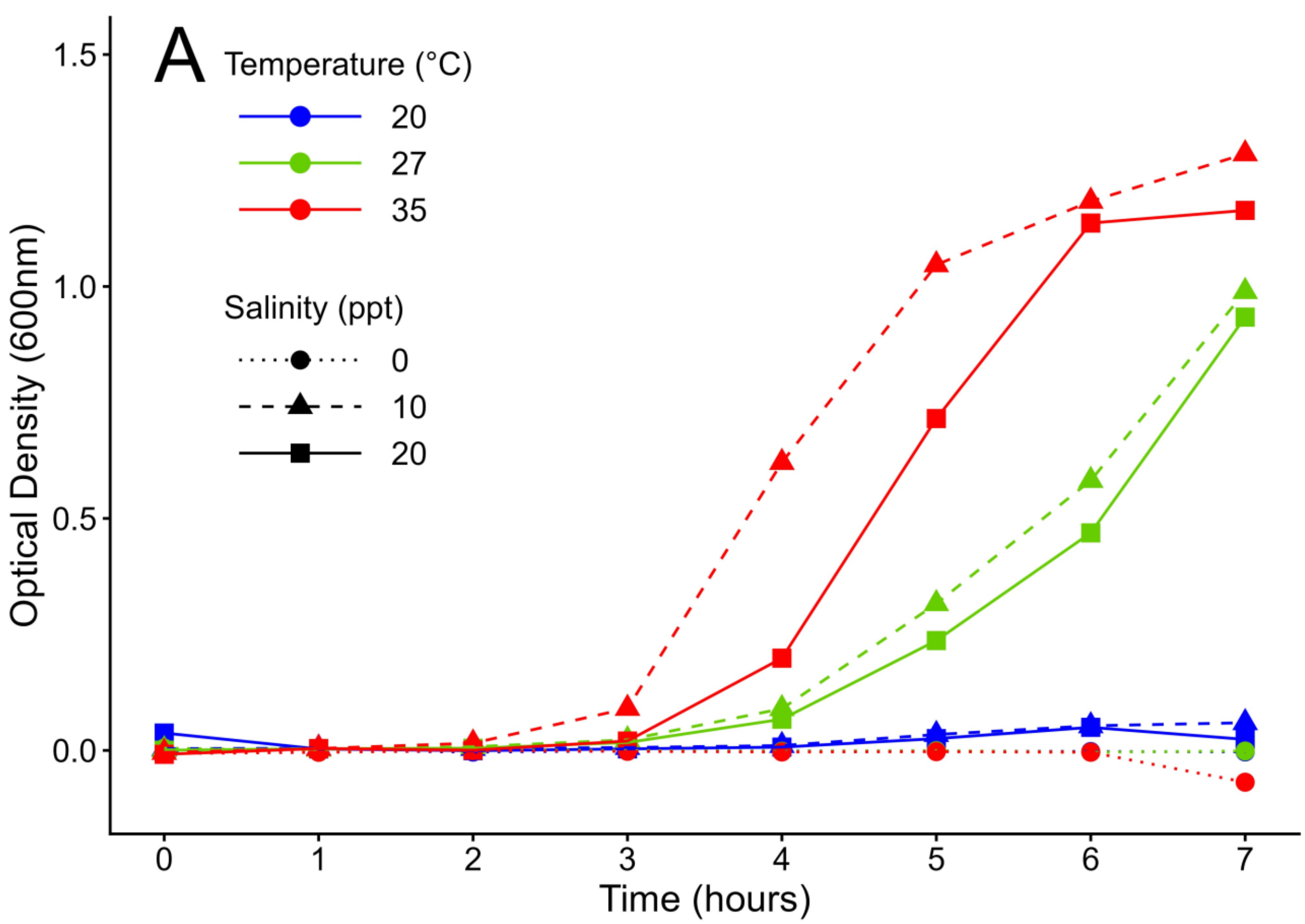

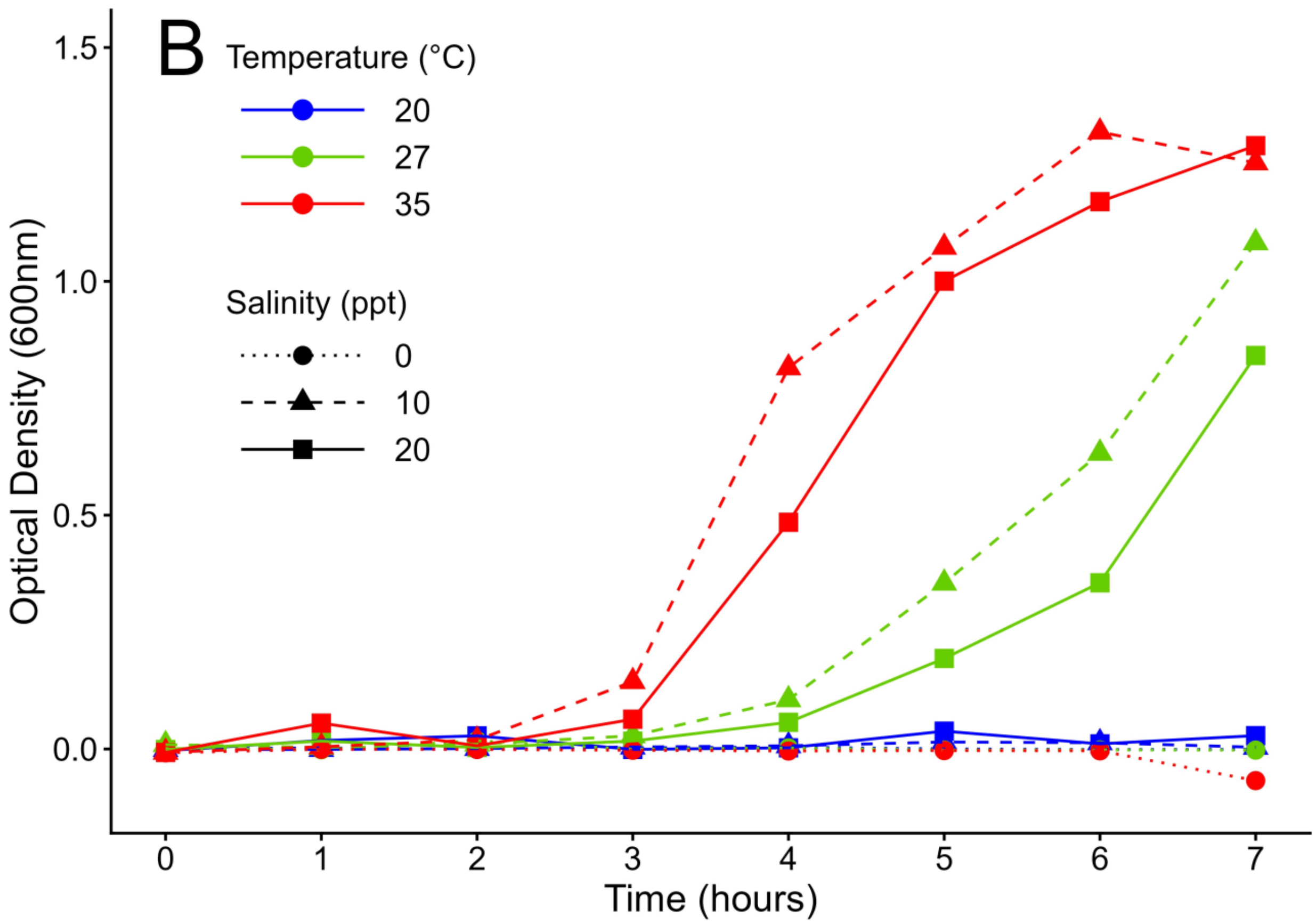

Fig. S2. In vitro growth kinetics of two alternate isolates of *V. vulnificus*. A) isolate AK-2020-515C and B) AK-2021-C-C. The bacteria were cultured at three different temperatures: 20°C (blue), 27°C (green), and 35°C (red) and three different salinities: 0 ppt (dotted line, circle), 10 ppt (dashed line, triangle) and 20 ppt (solid line, square). Growth of bacteria was measured hourly for 7 hours by optical density at 600 nm. Both isolates were recovered from eels showing clinical signs of RSD. Isolate AK-2020-515C was obtained in 2020 from a commercially harvested eel from the York River while isolate AK-2021-C-C was obtained in 2021 from an eel from an aquaculturist in Connecticut who mainly fished in Eastern Bay, a tributary of the Chesapeake Bay.
